# Recent advances in understanding extremophiles

**DOI:** 10.12688/f1000research.20765.1

**Published:** 2019-11-13

**Authors:** James A Coker

**Affiliations:** 1 Department of Sciences, University of Maryland Global Campus, Adelphi, MD, USA

**Keywords:** extremophile, acidophile, alkaliphile, halophile, psychrophile, thermophile, piezophile, astrobiology, biotechnology, radiophile, model organism, replication, TBP, TFB, TATA, 16S rRNA, PCB, PHA

## Abstract

Despite the typical human notion that the Earth is a habitable planet, over three quarters of our planet is uninhabitable by us without assistance. The organisms that live and thrive in these “inhospitable” environments are known by the name extremophiles and are found in all Domains of Life. Despite our general lack of knowledge about them, they have already assisted humans in many ways and still have much more to give. In this review, I describe how they have adapted to live/thrive/survive in their niches, helped scientists unlock major scientific discoveries, advance the field of biotechnology, and inform us about the boundaries of Life and where we might find it in the Universe.

## Introduction

As human beings, we tend to look at the world from our perspective and in turn the conditions under which our species thrives as “normal” and “typical” for the planet Earth; however, this is quite far from the truth. When looked at as a whole, the Earth is actually quite a cold place since 90% of the world’s oceans are not more than 5 °C ^1^. When the polar and alpine regions are factored in, cold environments account for roughly three quarters of the planet Earth. Therefore, in regard to extremophiles, it is important to understand that they are “extreme” only because they are found in regions where human beings typically cannot live unassisted. It is also important to think of them as growing and thriving in these conditions and not merely eking out an existence as the “last organism standing” that was able to survive just a bit longer than the rest. After all, they have evolved a set of adaptations that have made these “extreme” environments their preferred niche.

All three Domains of Life are represented in all of Earth’s extreme environments. However, a vast majority are prokaryotes, as they are the most numerous organisms on the planet, so it should come as no surprise that they have been isolated or detected literally all over the Earth. Just looking at the known limits of growth — minus 12 °C ( *Psychromoas ingrahamii*) to 129 °C ( *Geogemma barossii*), pH of less than 0 ( *Picrophilus torridus*) to 13 ( *Plectonema nostocorum* and *Hydrogenophaga* sp.), pressures of more than 100 Mpa ( *Shewanella benthica*), beyond saturating conditions of NaCl and KCl ( *Haloferax volcanii*), and high levels of ultraviolet (UV) (>100 J/m ^2^) and gamma (>12 kGy) radiation ( *Halobacterium* sp. NRC-1 and *Deinococcus radiodurans*) ^2– 8^—should more than confirm this. In addition to these gradients, viable microbes have been found from the mesosphere (48 to 77 km above the Earth’s surface) ^9^ to the Mariana Trench/Challenger Deep (11 km below the ocean surface) ^6^ to several kilometers below the Earth’s surface (for example, the South African gold mines) ^10^.

In the following review, I will catalog the adaptations that the organisms that live in these environments have evolved to persist and thrive. I will also describe a few major contributions made to the body of scientific knowledge that were made by using these organisms as model systems. I will finish by discussing two topics most often associated with extremophiles: biotechnology applications and astrobiology.

## Adaptations to environmental conditions

### pH: acidophiles

With few exceptions, these organisms maintain a cellular pH near neutral, which requires maintaining a pH gradient that is several orders of magnitude different on either side of the plasma membrane ^11^. One benefit of this is that there is always a ready supply of protons to power the proton motive force and form adenosine triphosphate (ATP) via the electron transport chain. However, the flip side is that the unusually high differences in concentration of protons (compared with neutrophiles) mean that, if left unchecked, the incoming protons have a greater capacity to dramatically change the internal cellular pH, which would lead to cell death.

To counteract these conditions, acidophiles have evolved several mechanisms. One of these is a cell membrane that is fairly impermeable to protons ^12^. In the Archaea branch of Life, this impermeability has been shown via tetraether lipids, differences in lipid head-group structures, a bulky isoprenoid core, and the fact that its ether linkages are less sensitive to acid hydrolysis compared with the ester linkages in the Bacteria and Eukarya Domains ^13^. Another mechanism is the reduced pore size of membrane channels, which has been shown for *Acidithiobacillus ferrooxidans* ^14^. Acidophiles also have a net positive potential charge inside the cell, which can counteract the high concentration of H ^+^ ions in their surroundings. They also employ active proton pumping as has been observed in *Bacillus* and *Termoplasma* species ^15^. It has not been shown that there is an identifiable acid response signature in the genomes of acidophiles. However, it is interesting to note that the genomes of acidophiles are predominately smaller than neutrophiles ^13^. It is currently unclear why this should be and whether it confers any evolutionary advantage to these organisms.

### pH: alkaliphiles

When adaptations are discussed, alkaliphiles are often grouped with halophiles, as they are typically found in saline environments ^16^. However, the response to high pH is specific to these organisms and is worth discussing. The cytoplasm of alkaliphiles, like that of acidophiles, is typically near neutral pH ^17^; therefore, alkaliphiles also have to overcome an imbalance of H ^+^ ions ^18^. Where acidophiles are “swimming” in H ^+^ ions, alkaliphiles are in a relative desert by comparison.

In response to this challenge, alkaliphiles have developed a negatively charged cell wall, which lowers the pH of the environment just outside the cell. They also produce an acidic secondary cell wall composed of teichurono-peptide and teichuronic acid or polyglutamic acid. These acids attract H ^+^ and repel OH ^−^, possibly helping to generate the proton motive force needed to drive ATP synthesis. In several alkaliphilic *Bacillus* species, the proton motive force for ATP synthesis is driven by Na ^+^ or K ^+^ antiporters that catalyse an electrogenic exchange of outwardly moving ions (Na ^+^ or K ^+^) and an increased number of entering H ^+^^19^. More generally, alkaliphiles are able to use these antiporters (Na ^+^/H ^+^ and K ^+^/H ^+^) ^20^ and also produce acids to reduce the internal pH when it is too high for metabolism to occur ^7^.

### Salinity: halophiles

Organisms that require a saline environment to grow (also known as halophiles) can be found along a continuum of salinity. These organisms have adopted either a “salt in” or “salt out” approach as the main adaptation for their ability to thrive in these conditions ^21^.

As suggested by the name, the salt-in approach means that these organisms have salinity/ion concentrations (up to 4 or 5 M) that are similar both inside and outside the cell membrane. As such, the cytosol of these organisms presents a significant challenge for the regular biochemistry of life. High-salinity conditions typically strip water molecules from proteins, resulting in aggregation and precipitation, and often this is due to exposed hydrophobic patches binding to one another. To counteract this, these organisms have evolved a proteome that is composed of primarily acidic proteins ^22^, and the acidic residues (aspartic and glutamic acid) are typically found on the surface of most of their proteins. These acidic residues have been shown to coordinate water molecules (that is, H ^+^ of water interacts with the COO ^−^ of the acidic side chain) around the proteins forming a “water cage” that protects the proteins from being dehydrated and precipitating out of solution ^23, 24^. As a result of the large-scale evolutionary changes needed for this survival (that is, changes to the proteome), these organisms tend to live mainly in environments where salinity does not dramatically fluctuate frequently. Thus far, only prokaryotes (bacteria and archaea) have been shown to adopt this strategy ^25^.

In contrast to salt-in organisms, the salt-out organisms have differing concentrations of salt/ions inside and outside the cell membrane (similar to H ^+^ with acido- and alkaliphiles). This strategy is more energy-intensive than the salt-in strategy ^16^, as these organisms actively accumulate ions and organic osmolytes (for example, glycine betaine, ectoine/5-hydroxyectoine, glucosylglycerol, and dimethylsulfoniopropionate ^26^) to maintain turgor pressure. The accumulated compatible solutes eventually can be released into the environment via mechanosensitive channels or used as an energy source during times of lower external salinity ^25^. As the salt-out approach requires fewer large-scale evolutionary changes, organisms that have adopted it are able to grow over a wider range of salinity.

### Radiation: radiophiles

Organisms’ responses to primarily two main types of radiation—ionizing (gamma) radiation and UV radiation—have been studied. Although on the surface it may seem that the same mechanisms of adaptation should be involved in both, there are, in fact, quite a few differences, which are probably due to the types of damage caused by each.

Ionizing radiation is responsible mainly for double-stranded breaks in the genome of organisms. However, it has also been shown to damage both proteins and lipids and induce persistent oxidative stress ^27^. Therefore, ionizing radioresistant organisms have developed all, or a combination of, the following strategies: novel and adaptive DNA repair mechanisms, antioxidant and enzymatic defense systems, and a condensed nucleoid. Fast and accurate repair of genomes is essential in surviving doses of ionizing radiation. This has been shown to be accomplished through the use of the nucleotide excision repair pathway ( *uvr*A1B), base excision repair pathway ( *ung* and *mut*Y), and homologous recombination pathway ( *rec*A, *ruv*A, *ddr*A, and *ppr*A) in *D. radiodurans* ^28^ and single-stranded binding proteins in *Halobacterium* sp. NRC-1 (Rfa-like genes) ^4^ and *D. radiodurans* (DdrB and SSB) ^29, 30^. In some especially sensitive species, protein damage causes death before double-stranded breaks start to form. It has been suggested that, especially for bacterial species, the role of protein damage in cell death due to ionizing radiation is underestimated. For example, *D. radiodurans* cells contain several oxidative stress prevention and tolerance mechanisms: cell cleaning through elimination of oxidized macromolecules, selective protection of proteins against oxidative damage, and suppression of reactive oxygen species production. A condensed nucleoid has also been shown to promote the efficiency/accuracy of DNA repair ^31^ and to limit the diffusion ^32^ of radiation-generated DNA fragments.

Unlike ionizing radiation where DNA damage is primarily double-stranded breaks, UV radiation damages DNA in more subtle ways through the formation of cyclobutene pyrimidine dimers (that is, thymine dimers) and pyrimidine (6-4) pyrimidone photoproducts (that is, 6-4 photo products). These two types of damage account for roughly 80% of photolesions induced by UV radiation ^33^. However, cyclobutene pyrimidine dimers are far more numerous and typically outnumber pyrimidine (6-4) pyrimidone photoproducts 3 to 1. To repair these DNA lesions, organisms use a combination of photoreactivation ( *phr*) genes, nucleotide excision repair ( *uvr*ABCD, *xpf*, and *rad*), base excision repair ( *mut*Y and *nth*), and homologous recombination ( *rec*A and *rad*A/51) ^33^. Additionally, organisms have evolved a suite of photoprotection devices to protect themselves from continual exposure to UV radiation. These include carotenoids, superoxide dismutases and hydroperoxidases, gene duplication via polyploidy, and genome composition (that is, reduction in the number of bipyrimidine sequences) ^33^. However, as in ionizing radiation, reactive oxygen species interference with normal metabolic processes is a more typical cause of cell death ^34^.

### Pressure: piezophiles

These organisms are typically found in deep lakes such as Baikal (1.6 km) and Tanganyika (1.5 km), the ocean, and subsurface communities. To have an idea of how much pressure is involved, one must remember that hydrostatic pressure increases roughly 10.5 kPa per meter depth while lithostatic (overburden) pressure increases about 22.6 kPa per meter. This means that microbes growing at the bottom of the Mariana Trench (10.9 km below the ocean surface) are subjected to 114.4 kPa of pressure but that those in the South African gold mines (3.5 km below the Earth’s surface) face 79.1 kPa.

With increasing pressures, membranes lose fluidity and permeability as lipids pack more tightly and enter a gel phase similar to what happens at cold temperatures. To counteract this, bacterial piezophiles have been shown to incorporate polyunsaturated and monounsaturated fatty acids ^11^ or phosphatidylglycerol and phosphatidylcholine instead of phosphatidylethanolamine ^35^. Little is known about the adaptations in their archaeal counterparts, but alterations in the amount of certain archaeols seem to be important ^36^. Other mechanisms of adaptation are generally not well known, but a few reports suggest that changes are not due to a specific group of genes/enzymes but rather are the result of an overall change in metabolism ^37– 39^ — similar to how the salt-in halophiles have evolved an acidic proteome to keep proteins soluble and active at high salinities rather than altering the expression of a few genes.

### Temperature: psychrophiles

As mentioned above, the Earth and the Universe are both predominately cold environments. At first glance, psychrophile should be easy to define (that is, as something that grows in the cold) and it has often been defined as an organism with an optimum temperature of less than 15 °C ^40^. However, this definition has multiple problems: it is arbitrary, does not account for eukaryotes, and treats cells as mere thermal units ^41^. Others have adopted the terms eurypsychrophile and stenopsychrophile to refer to a “broad” or “narrow” range of growth at low temperatures ^42^. Still, some believe that the use of these terms does not “push” researchers enough to search for “true” psychrophiles – those organisms able to grow well below 0 °C. As such, there have been attempts to classify psychrophiles as those organisms that grow below 5 °C and to introduce the term cryophile, defined as organisms that can grow below 0 °C ^43^. So, whichever term you prefer, it is important to remember that in our efforts to communicate, scientists often impose strict restrictions that Nature does not create or recognize.

Psychrophiles have been isolated from a variety of natural (for example, polar regions, frozen lakes, and winter soils) and man-made (for example, refrigerators and cooling vents) environments. Microbes are generally subject to the temperature of their environment and as a consequence must find ways to adapt to the limitation placed on them by temperature.

To compensate for the negative effects of cold temperatures, organisms have developed several physiological adaptations, including regulating the fluidity of their membranes, synthesizing temperature-related chaperones, and producing antifreeze molecules ^44^. Psychrophiles regulate membrane fluidity through an increase in the number of branched-chain or unsaturated fatty acids or a shortening of the length of the fatty-acyl chains or both. Molecular chaperones are used to aid in the refolding of proteins and affect the levels of protein synthesis ^45^. Compatible solutes are used as cryoprotectors to lower the freezing point of the cytoplasm ^46^ and possibly prevent aggregation/denaturation of proteins, stabilize membranes, and scavenge free radicles in cold conditions ^47^. To reduce the damage caused by forming ice crystals, they may also use antifreeze or ice nucleation proteins ^48, 49^. Antifreeze proteins act by binding seed ice crystals, thereby inhibiting their growth while ice nucleation proteins prevent the supercooling of water by ice crystal formation.

Structural proteins are also affected by temperature. Enzymes must overcome at least two obstacles in order to maintain activity at low temperatures: cold denaturation and slower reaction rates. Cold denaturation occurs at low temperatures because decreasing temperature results in more ordered water molecules surrounding a protein’s surface, which results in their being less associated with the protein and pushing the system equilibrium toward the unfolded state ^44^. The second problem for enzymes at low temperatures is slower reaction rates. According to the Boltzmann equation, reaction rates increase with increasing temperature and decrease two- to three-fold for every 10 °C decrease. Therefore, if cold-active enzymes are to have activities on par with their mesophilic counterparts, they must have developed structural changes to overcome these thermodynamic barriers ^50^.

### Temperature: thermophiles

All portions of thermophiles, as with psychrophiles, are constantly exposed to temperature; therefore, they have adapted all macromolecules (DNA, lipids, and proteins) and complexes (cell surface, ribosomes, RNA polymerase, and so on) to remain functional. Among the most studied aspects of adaptation for thermophiles are those found in proteins. Additional networks of hydrogen bonds, decreased length of surface loops, enhanced secondary structure propensity, higher core hydrophobicity, increased van der Waals interactions, ionic interactions, and increased packing density have all been shown to contribute to protein thermostability ^22^. It was more recently shown that, in addition to using the above mechanisms, archaeal cells use a structure-stabilization approach (that is, proteins are more compact than their mesophilic homologs) but that bacterial cells use a sequence-stabilization approach (that is, a small number of strong interactions) ^51^. Another well-studied aspect of adaptation is the lipid composition of thermophilic membranes. Certain organisms, like *Thermatoga maritima*, have novel/specific lipids (15, 16-dimethyl-30-glycerylox-triacontanedioic acid) ^11^. The ether-based lipids of archaea have also been shown to be resistant to hydrolysis at high temperatures. These are also found in meso- and psychrophiles and so are not a specific thermal adaptation. However, some thermophilic archaeal cells do contain a monolayer composed of a “fused lipid bi-layer” that has also been shown to resist hydrolysis at higher temperatures ^24^.

The DNA of thermophiles also has a thermal resistance in that it has positive supertwists added by reverse gyrase ^52^. Additionally, an increase in GC base pairs in specific regions (stem-loops) has been shown to stabilize DNA. Archaeal thermophiles also have histones that are closely related to the H2A/B, H3, and H4 core histone of eukaryotes. The binding of these histones has been shown to increase the melting temperature of DNA ^53^.

## Model organisms and major discoveries

Until very recently, a major drag on extremophile research was a lack of model organisms. Typically, after isolation, the genes of extremophilic organisms would be cloned and transformed into well-established model organisms like *Escherichia coli*. This was a necessary step to keep a forward momentum in research; however, it was also limiting as it allowed studies targeting only a small number of genes/proteins at a time and not the whole organism grown under *in situ* conditions. It also kept researchers from pushing limits as it meant that in-depth studies would always be relegated to genes/proteins from organisms that could “conform” to being studied in mesophilic model systems. Omics experiments have alleviated some of these problems; however, they cannot truly replace studies that need to be carried out *in vivo*. For these experiments, true model organisms that are extremophiles are needed. Fortunately, there are now model organisms for all extremophile groups mentioned in this review; they include *Leptospirillum ferriphilum* (acidophile) ^54, 55^, *Sulfolobus solfataricus* (acidophile and thermophile) ^56, 57^, *Natronomonas pharaonis* (alkaliphile and halophile) ^58, 59^, *Bacillus halodurans* (halophile) ^60, 61^, *H. volcanii* (halophile) ^62, 63^, *Halobacterium* sp. NRC-1 (halophile and radiophile) ^64, 65^, *Wallemia ichthyophaga* (halophile) ^66, 67^, *D. radiodurans* (radiophile) ^68, 69^, *Thermococcus barophilus* (piezophile) ^70, 71^, *Halorubrum lacusprofundi* (halophile and psychrophile) ^72, 73^, *Pseudoalteromonas haloplanktis* (psychrophile) ^74, 75^, *Thermococcus kodakarensis* (thermophile) ^76, 77^, and *Thermus thermophilus* (thermophile) ** ^78, 79^.

Other than detailed knowledge about the mechanisms that allow these organisms to thrive, studies of these model organisms have also led to some significant discoveries. One of these was the finding of a novel gene regulation mechanism found in *Halobacterium* sp. NRC-1 ^80^ that was also shown to exist in other archaeal cells with multiple TATA-binding proteins (TBPs) or transcription factor B (TFBs) or both ^81, 82^. This mechanism uses pairs of TBPs and TFBs to express/regulate specific sets of genes and is similar to a mechanism found in the Metazoa ^83, 84^. Another is the primacy of biological function (that is, proteome/lipidome) over information (that is, genome). Studies performed using *D. radiodurans* showed that cells can function for some time after losing their genome (that is, complete fragmentation of the genome by gamma irradiation); however, the same is not true for a cell that loses its proteome ^68^. Just recently, there were reports using *T. thermophilus* and other organisms suggesting that the 16S genes of prokaryotes, once thought to be species-specific and so used for decades for phylogenetic analysis, are promiscuous and horizontally transferred ^79^.

## Biotechnology applications

There have been four great success stories for the application of extremophiles and their products: the application of DNA polymerases from thermophiles to polymerase chain reaction, the use of thermophilic organisms/enzymes to produce biofuels, the use of acidophilic and thermophilic organisms/enzymes in biomining, and the use of halophilic organisms/enzymes to produce carotenoids ^85^. Extremophilic lipases/proteases have also been used extensively in detergents, specifically designed for cold water washing, and fine chemical synthesis ^86^.

In addition to the current applications, several other possibilities have been suggested over the years. These include using cold-active beta-galactosidase to hydrolyze lactose in dairy products ^87^ and using thermophilic and alkaliphilic starch-hydrolyzing enzymes (for example, alpha-amylase, glucoamylase, and pullulanase) to make a range of products like ethanol and high-fructose corn syrup ^85, 88^. Additionally, the products of extremophiles like sugars (for example, trehalose) and amino acid derivatives (for example, ectoine) as stabilizers for antibodies and vaccines or as skin care products ^24, 89^ have been suggested. Several extremophiles produce polyhydroxyalkanoates (PHAs), which are a heterogeneous group of polyesters and can be used to generate bioplastics ^90^. Finally, several extremophiles have been tested and shown to be helpful, at least on a small scale, in bioremediation efforts against heavy metals, radioactive isotopes, hydrocarbons, and polychlorobiphenyls (PCBs) to name a few ^91^.

## Astrobiology and origin-of-life theories

In addition to teaching us about the limits of life on Earth, extremophiles can tell us about the limits of life in the Universe. We should not expect that life, if found, in other parts of the Universe will necessarily be the same as on Earth; however, the laws of chemistry and physics we know suggest that life requires building blocks (for example, nucleotides and amino acids), an energy source (for example, solar radiation or redox reactions), and a liquid solvent (for example, water) ^92^. The known limits of life on Earth are mentioned in the introduction above; however, scientists keep finding exceptions and it is likely that the absolute limits, if they exist, have not been discovered. Indeed, the true limits of life are possibly the availability of water, building blocks, and an energy source as opposed to a specific temperature, pH, and so on ^93^.

Although extremophiles are often discussed by the predominate environmental pressure, in reality there are typically multiple extremes (for example, cold and high salinity or heat and acid). When considering these environments on Earth and other extraplanetary bodies, we must look increasingly toward polyextremophiles as the true model organisms for astrobiology ^94^.

At first blush, it might seem that the ever-extending boundary of life is a boon to detecting life on extraplanetary bodies. Indeed, that is the case but only to a limited extent. It is true that, as knowledge expands, we extend the types of places we expect to find life. However, one should remember that, with the exception of the Oceans, the environments where extremophiles thrive are quite far from a planetary mean ^95^. Additionally, organisms may be “active” (that is, metabolizing, dividing, and so on) only for brief moments in transient environments (for example, seasonal rains/water) ^96^. As such, many extraplanetary bodies that could support life are likely to be overlooked. It also means that “one-off” observations of extraplanetary bodies are highly unlikely to find signatures of life.

## Abbreviations

TBP, TATA-binding protein; TFB, transcription factor B; UV, ultraviolet
